# Egg Consumption and Blood Lipid Parameters According to the Presence of Chronic Metabolic Disorders: The EVIDENT II Study

**DOI:** 10.1210/clinem/dgab802

**Published:** 2021-11-03

**Authors:** Arthur Eumann Mesas, Miriam Garrido-Miguel, Rubén Fernández-Rodríguez, Sofía Fernández Franco, Cristina Lugones-Sánchez, Luis García-Ortiz, Vicente Martínez-Vizcaíno

**Affiliations:** 1 Universidad de Castilla-La Mancha, Health and Social Research Center, Cuenca, 16071, Spain; 2 Universidade Estadual de Londrina, Londrina, Paraná, 86057-970, Brazil; 3 Universidad de Castilla-La Mancha, Faculty of Nursing, Albacete, 02006, Spain; 4 Grupo Avícola Rujamar, San Lorenzo de la Parrilla, Cuenca, 16770, Spain; 5 Unidad de Investigación en Atención Primaria de Salamanca (APISAL), Instituto de investigación Biomédica de Salamanca (IBSAL), Gerencia de Atención Primaria de Salamanca, Gerencia Regional de Salud de Castilla y León (SACyL), Salamanca, 37005, Spain; 6 Universidad de Salamanca, Departamento de Ciencias Biomédicas y del Diagnóstico, Salamanca, 37007, Spain; 7 Investigadores grupo EVIDENT, redIAPP: Red Española de Investigación para Actividades Preventivas y Promoción de la Salud en Atención Primaria; 8 Universidad Autónoma de Chile, Facultad de Ciencias de la Salud, Talca, 1101, Chile

**Keywords:** eggs, cholesterol, lipids, dyslipidemia, high-density lipoprotein cholesterol, comorbidity

## Abstract

**Context:**

Egg consumption is one of the main dietary sources of cholesterol, but whether individuals who eat more eggs have a worse blood lipid profile remains controversial.

**Objective:**

We examined the relationship between egg consumption and lipid parameters and explored whether this relationship changes according to the presence of chronic metabolic disorders.

**Methods:**

A multicenter cross-sectional study was conducted with adult participants in the EVIDENT II trial. Adjusted linear regression models were stratified by the main chronic metabolic disorders.

**Results:**

Among the 728 participants (61.9% women, mean age 52.1 ± 11.9 years), the mean egg consumption was equivalent to 5 to 6 eggs per week for a 70-kg individual. In the fully adjusted analysis, no association was found of egg consumption with total and high-density lipoprotein cholesterol (HDL-c), and triglyceride levels. Furthermore, compared with the first quartile of consumption, the fourth quartile was associated with lower low-density lipoprotein cholesterol (LDL-c) levels (coefficient –7.01; 95% CI –13.39, –0.62) and a lower LDL-c/HDL-c ratio (coefficient –0.24, 95% CI –0.41, –0.06). In the analyses stratified by chronic metabolic diseases, higher egg consumption was not associated with lipid profile in those with obesity, hypertension, type 2 diabetes, dyslipidemia, or treated with hypolipidemic drugs, and was associated with a better lipid profile in participants without these conditions.

**Conclusion:**

Higher egg consumption was not associated with blood lipids in individuals with chronic metabolic disorders. In individuals without such conditions, the lipid profile was better among those who consumed more eggs. Our findings support current guidelines recommending eggs as part of a healthy diet.

Eggs are a food with good nutritional density and are excellent sources of animal protein and bioactive nutrients (1). However, egg yolk is one of the main sources of dietary cholesterol (2), that has been inconsistently associated with increased cardiovascular risk (3-5). The increased cardiometabolic risk due to high egg consumption could be through increased total blood lipids. However, the debate about whether the blood lipid profile is worse in those who consume eggs on a regular basis has been ongoing for decades, and no definitive conclusion has yet been reached (6, 7).

The association between egg consumption and blood lipid parameters has been explored in several meta-analyses of randomized controlled trials (RCTs) (8, 9), and the results were contradictory. The first meta-analysis including 17 RCTs reported that dietary cholesterol (including that from eggs) raises the ratio of total to high-density lipoprotein cholesterol (HDL-c) and, therefore, adversely affects the cholesterol profile (10). A recent updated meta-analysis based on 66 RCTs also reported a linear correlation between the consumption of greater than 1 egg per day in a short time period (no long time periods) and worsening lipid profiles (7). Similar results were found in another meta-analysis with 17 RCTs in a healthy population (11). Otherwise, nondifferential effects in blood pressure, lipid levels and lipoprotein levels were observed in 8 RCTs comparing the intake of >4 whole eggs/week with ≤4 whole eggs/week (9). Last, in a meta-analysis of 28 RCTs, egg consumption increased total cholesterol (TC), low-density lipoprotein cholesterol (LDL-c) and HDL-c levels but not the LDL-c/HDL-c ratio, TC:HDL-c ratio or triglyceride (TG) levels compared with low egg consumption diets (8). In addition to the inconsistencies observed in RCTs, the results from observational studies are also inconclusive. For instance, it has been found that blood lipid levels do not differ (12) or are even lower (13) among higher egg consumers.

Considering that the lipid profile is closely associated with chronic cardiometabolic disorders such as obesity, hypertension, or type 2 diabetes (T2D) (14), it seems important to examine whether the effect of egg consumption on the lipid profile depends on the presence of these disorders. This analysis could be clinically relevant because, although the scientific evidence is not entirely consistent regarding this, the restriction of egg consumption is still recommended in most dietary guidelines (15), specifically those focused on dyslipidemia management (16). There appear to be 2 different patterns of association between egg consumption and cardiometabolic risk according to the characteristics of the population that is studied. On the one hand, studies with patients with hypertension (17), T2D (18), and dyslipidemia (17, 19) have found mixed results regarding blood lipid levels in patients who consumed eggs more frequently. However, studies with healthy patients without these conditions either found no significant association (20) or even observed a lower risk associated with higher egg consumption (13). Likewise, because these associations could be confounded by background dietary cholesterol issues (20) and other lifestyle-related behaviors, such as physical activity, quality of diet, tobacco, and alcohol intake (21), these factors should be controlled for in the corresponding analyses.

Therefore, this study examined the relationship between egg consumption and blood lipid parameters and focused on whether this relationship varies according to the presence of chronic metabolic disorders such as obesity, hypertension, T2D, and dyslipidemia.

## Material and Methods

### Study Design and Participants

This was a cross-sectional analysis of data from baseline measurements of the EVIDENT II trial (NCT02016014), a multicenter, randomized, double-blind clinical trial that aimed to develop and validate a smartphone application and to evaluate the effect of adding this tool to a standardized intervention designed to improve adherence to the Mediterranean diet and to physical activity (22). The study included 6 groups of the Research Network on Preventive Activities and Health Promotion (REDIAPP) in Bilbao, Cuenca, Zaragoza, Valladolid, Barcelona, and Salamanca (Spain). This trial included adult men and women (aged 18-70 years) free of advanced cardiovascular disease, cancer, and other major physical or mental disorders. Face-to-face and individual interviews, as well as anthropometric measurements, were performed in a research center by previously trained investigators. Recruitment, data collection, and measurement procedures have been described elsewhere (22). The study was approved by the Ethics Committee of Salamanca University Hospital (Spain), and all participants gave written informed consent according to the general recommendations of the Declaration of Helsinki.

Of the 833 participants who were initially examined, 30 were excluded because of a lack of dietary data, 20 were excluded because of a lack of biochemical lipid data, and 8 were excluded because of missing data on any of the covariates that were considered. Thus, the present analyses were based on a subsample of 728 individuals (85%) in which all dataset variables were measured. Participants did not differ in age, sex, or socioeconomic status from the whole sample.

### Study Variables

#### Exposure

Egg consumption was obtained with a 137-item Food Frequency Questionnaire that has been validated in a population of elderly people at high cardiovascular risk in Spain (23). An incremental scale with 9 levels, from “never or almost never” to “>6 times/day”, was used to collect information on food consumption frequencies. Because body weight (BW) is an important variable to consider when studying the relationship between the number of eggs ingested and the serum lipid profile, we used g/day/kg body weight as the unit of measurement, the same currently used by the European Food Safety Authority in recommended dietary guidelines (24, 25).

#### Outcomes

The dependent variables included the following serum lipid parameters: TC, TG, HDL-c, and LDL-c levels. These biochemical profiles were measured by enzymatic methods (Boehringer Mannheim Corporation, Mannheim, Germany). Blood samples were obtained from the cubital vein between 8.00 and 9.00 am after the individuals had fasted and abstained from smoking, alcohol, and caffeinated beverages for the previous 12 hours. Blood samples were collected at the respective health centers, and all samples were analyzed at the city hospital that participates in the external quality assurance programs of the Spanish Society of Clinical Chemistry and Molecular Pathology.

#### Covariates

Information was also collected on potentially confounding covariates of the association between egg consumption and serum lipid levels, including sociodemographic variables (age, sex, educational level) and body mass index (BMI) obtained by objective measures of height divided by height squared, total energy intake (in kcal/day, obtained with the 137-item Food Frequency Questionnaire), adherence to the Mediterranean diet (obtained with the validated 14-point Mediterranean Diet Adherence Screener (26), tobacco consumption (yes or no), alcohol intake (yes or no), and leisure time physical activity (in METS-min/week, measured with the International Physical Activity Questionnaire). Finally, participants were asked whether they had been diagnosed with hypertension, T2D, or dyslipidemia and whether they used lipid-lowering drugs. As a summary variable, participants identified as having ≥1 chronic condition were considered as having obesity (BMI ≥30 kg/m^2^), hypertension, T2D, dyslipidemia, and/or using lipid-lowering drugs.

### Statistical Analysis

To differentiate between the highest and lowest egg consumption, quartiles of consumption were established in the statistical analyses. The group with the lowest intake category (up to 1 egg/week) was used as the reference category. To give a more applicable sense of the unit of measurement of egg consumption, if an individual with a BW of 70 kg and a standard egg weight of 60 g is considered, the first quartile varies from 0 eggs to 1 egg consumed per week (0.14 g/day/kg BW = 0.14 × 70 kg × 7 days/60 g = 1.14 or approximately 1 egg/week). The same calculation shows that the last quartile, with the highest category of consumption, comprises the intake of >3 eggs per week. Of the 230 participants classified in the last quartile, only 4 participants consumed more than 7 eggs per week (maximum of 18 eggs per week consumed by an individual).

Statistical analysis included a description of the study population and the variables analyzed in total and by quartiles of egg consumption. First, the normal distribution of continuous variables was examined using both statistical (Kolmogorov–Smirnov test) and graphical (normal probability plots) methods. After that, chi-squared tests were used for categorical variables, and analysis of variance was used for continuous variables to compare the mean differences of each variable according to the categories of egg consumption. Pairwise multiple comparisons were examined using the post hoc Bonferroni test. Then, the Pearson correlation test was applied considering the continuous variables of egg intake (g/day/kg of BW), TC levels, LDL-c levels, HDL-c levels, the LDL-c/HDL-c ratio, and TG levels.

Linear regression models were used to analyze the association between egg consumption (independent variable) in consumption quartiles and continuous consumption (to explore the possible trend of change in lipid levels according to the continuous increase in egg consumption) with each of the serum lipid parameters (dependent variable). The first adjusted model included age, sex, education level, smoking status, alcohol intake, total energy intake, adherence to the Mediterranean diet, and free-time physical activity as potential confounding variables. In a second model, obesity, hypertension, T2D, dyslipidemia, and use of hypolipidemic drugs were also included.

Finally, to explore whether the associations of interest changed according to the chronic conditions studied, the models with complete adjustment were stratified by the presence or absence of obesity, hypertension, T2D, dyslipidemia, use of lipid-lowering drugs, and by presenting ≥1 of those conditions.

All analyses were carried out with the STATA program (version 15), and *P* < .05 was considered statistically significant.

## Results

Among the 728 participants studied (61.9% women, mean 52.1 ± 11.9 years of age), the mean egg consumption was 0.30 ± 0.16 g/day/kg of BW, equivalent to 5 to 6 eggs per week for a 70-kg individual. As observed in Table 1, the consumption of eggs was higher in women, particularly in those with a lower BMI and with a higher total caloric intake. Regarding blood lipid parameters, TC and LDL-c levels were higher in the third quartile of egg consumption than in the other quartiles. In addition, HDL-c levels were higher, and TG levels and the LDL-c/HDL-c ratio were lower in the quartile with the highest egg consumption (fourth quartile). Finally, among the individuals with the highest egg consumption, a lower frequency of obesity, hypertension, dyslipidemia, and ≥1 chronic condition was observed.

**Table 1.** Characteristics of the study participants by the number of eggs consumed per week per kg of body weight

Characteristic	Total	Egg consumption (g/day/kg BW)				*P* value*
		First quartile (0-0.14)	Second quartile (>0.14-0.30)	Third quartile (>0.30-0.39)	Fourth quartile (>0.39)	
Number of 60 g eggs/week for an individual with a BW of 70 kg		0-1	>1-2.5	>2.5-3	>3	
Total, n (%)	728 (100.0)	176 (24.2)	180 (24.7)	189 (26.0)	183 (25.1)	
Age (years)	52.1 ± 11.9	51.8 ± 12.5^a^	51.1 ± 11.4^a^	55.8 ± 10.1^b^	49.6 ± 12.6^a^	**<.001**
Female, n (%)	451 (61.9)	95 (54.0)	88 (48.9)	118 (62.4)	150 (82.0)	**<.001**
University studies	216 (29.7)	46 (26.1)	57 (31.7)	52 (27.5)	61 (33.3)	.39
Body mass index (kg/m^2^)	27.8 ± 4.8	28.6 ± 4.4^a^	30.3 ± 5.9^b^	27.7 ± 2.7^a^	24.8 ± 4.1^c^	**<.001**
Smoker, n (%)	143 (19.6)	34 (19.3)	37 (20.6)	32 (16.9)	40 (21.9)	.67
Alcohol drinker, n (%)	572 (78.6)	133 (75.6)	145 (80.5)	152 (80.4)	142 (77.6)	.60
Physical activity (METS-min/week)	455 ± 214	444 ± 198	429 ± 215	479 ± 228	466 ± 213	.11
Total energy (kcal/day)	2469 ± 779	2217 ± 869^a^	2521 ± 761^b^	2475 ± 632^b^	2655 ± 784^b^	**<.001**
Lipid profile (mg/dL)						
TC level	203.9 ± 34.8	201.6 ± 35.2^a^	201.9 ± 33.9^a^	212.8 ± 35.4^b^	198.8 ± 33.4^a^	**<.001**
LDL-c level	124.7 ± 31.3	124.3 ± 32.2^a^	122.9 ± 30.2^a^	132.3 ± 31.2^b^	118.8 ± 30.4^a^	**<.001**
HDL-c level	59.0 ± 15.1	55.6 ± 13.9^a^	56.8 ± 16.0^a^	60.2 ± 14.4^b^	63.3 ± 14.7^b^	**<.001**
TG level	107.9 ± 56.1	116.9 ± 67.3^a^	116.2 ± 54.6^a^	108.2 ± 49.9^a^	90.5 ± 47.5^b^	**<.001**
LDL-c/HDL-c ratio	2.3 ± 0.9	2.4 ± 0.9^a^	2.3 ± 0.8^a^	2.3 ± 0.8^a^	2.0 ± 0.8^b^	
TC level ≥200, n (%)	407 (55.9)	93 (52.8)	97 (53.9)	128 (67.7)	89 (48.6)	**.001**
LDL-c level ≥130, n (%)	306 (42.0)	75 (42.6)	72 (40.0)	102 (54.0)	57 (31.2)	**<.001**
HDL-c level <50 (M) or <40 (F), n (%)	134 (18.4)	35 (19.9)	39 (21.7)	33 (17.5)	27 (14.8)	.35
TG level ≥150, n (%)	125 (17.2)	32 (18.2)	41 (22.8)	33 (17.5)	19 (10.4)	**.018**
Comorbidity, n (%)						
Obesity	209 (28.7)	56 (31.8)	93 (51.7)	41 (21.7)	19 (10.4)	**<.001**
HTA	238 (32.7)	58 (33.0)	78 (43.3)	67 (35.5)	35 (19.1)	**<.001**
T2D	51 (7.0)	18 (10.2)	14 (7.8)	9 (4.8)	10 (5.5)	.165
Dyslipidemia	282 (38.7)	65 (36.9)	76 (42.2)	89 (47.1)	52 (28.4)	**.002**
Use of hypolipidemic drugs	144 (19.8)	38 (21.6)	38 (21.1)	43 (22.8)	25 (13.7)	.115
≥1 chronic condition	470 (64.6)	115 (65.3)	146 (81.1)	128 (67.7)	81 (44.3)	**<.001**

Values are means ± SD. *P* values marked with bold indicate statistically significant differences between the quartiles of egg consumption.

Abbreviations, BW, body weight; F, female sex; HDL-c, high-density lipoprotein cholesterol; HTA, hypertension; LDL-c, low-density lipoprotein cholesterol; M, male sex; SD, standard deviation; T2D, type 2 diabetes; TC, total cholesterol; TG, triglyceride.

*Chi-squared test for categorical variables and analysis of variance for continuous variables. Different letters indicate significant differences identified with the Bonferroni post hoc test between quartiles of egg consumption.

As shown in Table 2, the consumption of eggs was positively correlated with HDL-c levels (r = 0.11) and the LDL-c/HDL-c ratio (r = –0.10) and negatively correlated with TG levels (r = –0.10). However, no statistical significance was found between egg consumption and TC and LDL-c levels.

**Table 2.** Bivariate correlation between egg consumption and blood lipid parameters

Variables	Egg intake	TC level	LDL-c level	HDL-c level	LDL-c/HDL-c ratio	TG level
Egg intake	1.00					
TC level	–0.001	1.00				
LDL-c level	–0.02	0.89 **	1.00			
HDL-c level	0.11 **	0.26 **	–0.06	1.00		
LDL-c/HDL-c ratio	–0.10 **	0.43 **	0.69 **	–0.69 **	1.00	
TG level	–0.10 **	0.20 **	0.1**	–0.45 **	0.45 **	1.00

Values indicate the correlation coefficient (r).

Abbreviations: HDL-c, high-density lipoprotein cholesterol; LDL-c, low-density lipoprotein cholesterol; TG, triglyceride; TC, total cholesterol.

*P < .05, **P < .001. Egg intake is in g/day/kg of body weight unit, and all lipid parameters are in mg/dL.

Table 3 shows the results of the association analyses. In the crude model, the third quartile of consumption (third vs first quartile) was associated with higher levels of TC, LDL-c, and HDL-c, while the fourth quartile was associated with higher HDL-c levels and with a lower LDL-c/HDL-c ratio and lower TG levels. All these associations were maintained after adjusting for sociodemographic variables, lifestyle, and BMI. However, in the fully adjusted results, no association was observed between egg consumption and TC, HDL-c, and TG levels. Furthermore, the fourth quartile (compared with the first quartile) was associated with lower LDL-c levels (coefficient –7.01; 95% CI –13.39, –0.62) and a lower LDL-c/HDL-c ratio (coefficient –0.24, 95% CI –0.41, –0.06).

**Table 3.** Linear regression models of the association between egg consumption and blood lipid parameters

Models	TC	LDL–c	HDL–c	LDL–c/HDL–c ratio	TG
Unadjusted model					
First quartile	Reference	Reference	Reference	Reference	Reference
Second quartile	0.27 (–6.90, 7.45)	–1.43 (–7.88, 5.02)	1.19 (–1.88, 4.27)	–0.07 (–0.24, 0.10)	–0.75 (–0.12, 10.73)
Third quartile	11.2 (4.04, 18.2)**	8.01 (1.63, 14.38)*	4.53 (1.49, 7.57)**	–0.06 (–0.23, 0.11)	–8.72 (–20.07, 2.62)
Fourth quartile	–2.82 (–9.97, 4.32)	–5.53 (–11.96, 0.88)	7.71 (4.65, 10.78)**	–0.39 (–0.56, –0.22)**	–26.43 (–37.87, –14.99)**
*P* value for trend	0.62	0.38	0.079	0.043	0.094
Model 1					
First quartile	Reference	Reference	Reference	Reference	Reference
Second quartile	0.61 (–6.50, 7.72)	–1.41 (–7.89, 5.05)	1.81 (–1.02, 4.65)	–0.08 (–0.26, 0.08)	–2.42 (–13.50, 8.66)
Third quartile	7.68 (0.56, 14.81)*	5.76 (–0.72, 12.24)	2.70 (–0.13, 5.55)	–0.02 (–0.19, 0.15)	–4.74 (–15.83, 6.35)
Fourth quartile	–5.90 (–13.29, 1.48)	–6.66 (–13.38, 0.05)	4.13 (1.18, 7.08)**	–0.28 (–0.45, –0.10)**	–15.33 (–26.83, –3.83)**
*P* value for trend	0.33	0.28	0.63	0.18	0.63
Model 2					
First quartile	Reference	Reference	Reference	Reference	Reference
Second quartile	–0.59 (–7.34, 6.23)	–3.07 (–9.27, 3.13)	3.14 (0.32, 5.96)*	–0.16 (–0.33, 0.01)	–8.23 (–19.13, 2.66)
Third quartile	3.79 (–2.92, 10.51)	2.59 (–3.55, 8.73)	1.90 (–0.88, 4.69)	–0.05 (–0.22, 0.12)	–4.02 (–14.81, 6.76)
Fourth quartile	–6.91 (–13.89, 0.08)	–7.01 (–13.39, –0.62)*	2.81 (–0.08, 5.71)	–0.24 (–0.41, –0.06)**	–11.09 (–22.32, 0.12)
*P* value for trend	0.23	0.21	0.99	0.26	0.95

Values indicate the coefficient (95% CI) obtained through linear regression models.

Model 1: adjusted for age, sex, education level, smoking status, alcohol intake, total energy intake, quality of diet and moderate-to-vigorous physical activity. Model 2: Model 1 adjusted for obesity, hypertension, type 2 diabetes, dyslipidemia and use of hypolipidemic drugs.

Abbreviations, HDL-c, high-density lipoprotein cholesterol; LDL-c, low-density lipoprotein cholesterol; TG, triglyceride; TC, total cholesterol.

*P < .05,**P < .001.

**Figure 1.**
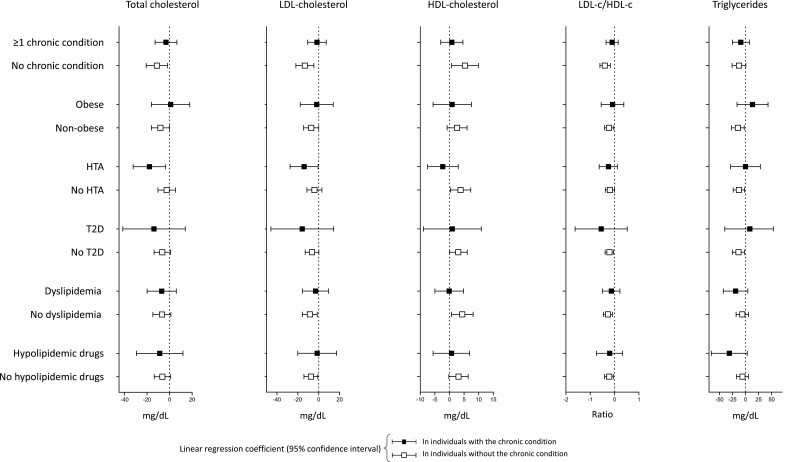
Linear regression coefficient (95% CI) of the fourth quartile vs the first quartile of egg consumption (independent variable) and blood lipid parameters (dependent variable) by the presence of ≥1 chronic condition, obesity, hypertension (HTA), type 2 diabetes (T2D), dyslipidemia, and use of hypolipidemic drugs. All values were adjusted for the following variables (except for the stratification variable itself): age, sex, education level, smoking status, alcohol intake, total energy intake, quality of diet, moderate-to-vigorous physical activity, obesity, HTA, T2D, dyslipidemia and use of hypolipidemic drugs.

Finally, in the analyses stratified by chronic metabolic diseases shown in Fig. 1, in general, higher egg consumption was associated (fourth vs first quartile) with a better lipid profile in individuals without metabolic diseases and was not associated with serum lipid parameters in individuals with obesity, hypertension, T2D, dyslipidemia, and/or treated with hypolipidemic drugs.

## Discussion

In this cross-sectional study, egg consumption was not associated with TC, HDL-c, or TG levels in analyses controlling for the main potential confounders, including chronic metabolic comorbidities. Furthermore, higher egg consumption was independently associated with lower LDL-c levels and LDL-c/HDL-c ratio. In the analyses stratified by chronic metabolic conditions, individuals without comorbidities who consumed >3 eggs per week were more likely to present a better lipid profile than those who consumed up to 1 egg per week.

Our results for the absence of an association between egg consumption and blood lipid parameters are consistent with some observational studies, including cross-sectional (13, 27-29) and longitudinal (12, 30) studies carried out with adult populations. Furthermore, when controlling for the potential confounding effect of a series of covariates for lifestyle and chronic conditions, our findings regarding no association remained stable, which indicates that these conditions have only a partial confounding effect. Nevertheless, because in our population the average egg consumption was moderate and differences in plasma lipids were observed by comparing values in a narrow range of weekly egg consumption, it cannot be ruled out that egg consumption did not correlate with LDL-c or was not associated with TC, HDL-c, and TG in the regression models because of insufficient statistical power to detect statistical significance in small differences.

To the best of our knowledge, no observational study has reported worse blood lipid parameters in individuals reporting higher egg consumption. However, both in prospective cohorts (31-33) and in a meta-analysis of cohort studies (34), egg consumption was associated with increased incident cardiovascular disease and all-cause mortality, mainly in people with T2D. A reason for this could be that there were people with obesity, hypercholesterolemia, or hypertriglyceridemia among the participants, in which blood lipid levels may have been affected by their background lipid levels (20).

In addition to confirming the absence of an association between egg consumption and the blood lipid profile found in previous studies (12, 27), our results indicate a possible beneficial influence on the lipid profile in healthy individuals who consume more eggs. This apparently paradoxical beneficial effect observed in healthy people (ie, improving the lipid profile by consuming more eggs, a food rich in lipids) could be due to a healthier lifestyle, based on a better quality of diet, lower total energy intake, or higher levels of physical activity (21, 35). In other words, the better lipid profile observed in healthy individuals who consume more eggs could be partially explained by the fact that they consume more protein, have more lean mass, and are physically more active, which results in lower endogenous lipid production and optimizes plasma lipid metabolism. However, the confounding effect that the mentioned variables could cause is possibly small, since the main results remained practically unchanged after adjusting the models for the confounding variables. It is also known that not all cholesterol consumed in the diet is digested and absorbed by the body (36), and in eggs, it is possibly due to the phospholipids that are present (37). Furthermore, it has been seen that these lipid blood levels depend more on the intake of saturated fatty acids than on dietary cholesterol (31, 38). Although eggs are one of the main sources of dietary cholesterol (a large 60-g egg contains ~230 mg of cholesterol (2)), they also contain other bioactive components that may modify the body’s response to the dietary cholesterol found in eggs, such as phospholipids, amino acids (ie, glycine, methionine, cysteine), and mono- and polyunsaturated fatty acids (39). In this sense, future studies are needed to examine the potential benefits of these other components in eggs on the blood lipid profile and cardiometabolic risk.

The main limitation of our study is the cross-sectional design, which does not support the claim that long-term egg consumption does not change or even improve the lipid profile. In addition, reverse causality cannot be excluded; that is, people without chronic metabolic disorders and, therefore, with a better lipid profile may consume more eggs because they are aware that they have a low risk of increasing their blood cholesterol. On the other hand, underlying metabolic disorders are also associated with plasma lipid levels, so plasma cholesterol may be associated with the disease itself regardless of the consumption of eggs. For example, individuals with T2D are less likely to have high LDL-c (40), and, therefore, low plasma levels of these lipoproteins would not necessarily be related to the consumption of eggs. Another limitation is that the information on diet was collected with a questionnaire, which is subject to recall bias and information bias due to the greater health consciousness of those without chronic metabolic disorders (41). In addition, it is also important to emphasize that the way in which the consumed eggs were prepared was not differentiated; that is, if they were eaten cooked or fried and, if they were fried in what type and quantity of oil. However, we must consider that the results of our study add value to the available evidence by being one of the first studies to weigh the consumption of eggs per kg of BW, a more suitable unit of measure to assess food consumption, especially its relationship with biochemical and metabolic parameters. Additionally, it is known that a higher intake of saturated fat would have a greater effect on plasma lipids and could confuse the association between egg consumption and lipid profile. Although our analyses did not control specifically for saturated fat consumption, they controlled for an indicator of diet quality based on adherence to the Mediterranean diet, which considers the lower frequency of consumption of saturated fats and higher frequency of unsaturated fat–rich foods such as olive oil and nuts (42). Moreover, although the acute individual change in plasma total cholesterol following dietary cholesterol intake, as well as the resulting classification of participants into normal-hypo- or hyper-responders might help to understand the association between egg consumption and plasma lipoproteins (43), this information was not obtained in this study. However, the risk of developing an atherogenic lipoprotein profile in healthy men was not increased after consumption of additional dietary cholesterol from eggs, regardless of hypo- or hyper-responder classification (44). Additionally, as a positive aspect of this study, the analyses were adjusted for the main sociodemographic, anthropometric, and lifestyle confounders, although the possibility of specific residual confounders, such as ethnicity, marital status, or physical fitness, should always be considered.

### Conclusion

In conclusion, according to our results, the consumption of >3 eggs per week by an adult with a BW of 70 kg is associated with a 7 mg/dL decrease in LDL-c mg/dL levels and a reduced LDL-c/HDL-c ratio compared with the consumption of up to 1 egg per week. In addition, in both adults with and without chronic comorbidities, such as hypertension, diabetes, and dyslipidemia, lower TC and higher HDL-c levels were observed among adults who consumed more eggs than among those who consumed fewer eggs. Although these results must be confirmed in prospective studies that adjust for total caloric intake, quality of diet, and physical activity, this study reinforces the evidence in favor of the current guidelines that recommend the maintenance of moderate egg consumption as part of a balanced diet (15, 45). Finally, our findings have relevant clinical implications because they support the lack of evidence for the recommendation contained in clinical guidelines from some years ago to restrict egg consumption, both in healthy adults and adults with cardiometabolic disorders such as hypertension, T2D, and dyslipidemia.

## Data Availability

The datasets generated during and/or analyzed during the current study are available from the corresponding author (Miriam Garrido-Miguel) on reasonable request.

## Abbreviations

BMI: body mass index
BW: body weight
LDL-c: low-density lipoprotein cholesterol
HDL-c: high-density lipoprotein cholesterol
RCT: randomized controlled trial
TC: total cholesterol
T2D: type 2 diabetes
TG: triglyceride
